# Completion of Volume VI

**Published:** 1867-07

**Authors:** 


### Completion of Volume VI.

With the present number we complete six years of editorial life. Looking over its results, we find some successes with which to be gratified, and feel that there are imperfections and failures to be studiously avoided in future. We have endeavored to conduct the Journal with strict impartiality in the true interest of the whole profession, and that we have not failed in this purpose is demonstrated by the constantly increasing and steadfast support it has received at its hands. We have to thank the true, progressive, intelligent and earnest members of our profession, within the circle of our influence, for the successes thus far achieved, and to acknowledge that it has depended mainly upon them. We are happy to believe, that almost without exception, the physicians of Western New York, and of the adjoining portions of other States, as well as Canada, have taken sufficient interest in this enterprise, to aid it at least by the amount of the yearly subscription, while many have generously and ably contributed to its pages. It was the only medical monthly periodical in the State, not suspended during the financial disturbances of the war, and now stands upon a permanent basis, open to all true, honest observers, as medium of professional communication. It has been the purpose to furnish original, suggestive and practical matter, and to avoid as much as possible the crude, undigested, uncertain and worthless; and to this task, we have devoted the time which the duties of active practice would permit, extracting however from the period usually devoted to repose, the major part. If the Journal has sometimes suffered from neglect, those who understand these circumstances best, will be the most ready to excuse the defect, while only the disappointed, unsatisfied, unhappy grumblers in the profession, who make no progress themselves, and dread advancement in others, will be found ready to underrate its value. Competent and impartial readers have everywhere manifested appreciation of the value of its original observations, and the verdict of the profession has already sustained the enterprise. But we desire to introduce a plea for the Journal in future, and leave the past for history.

Will all the present readers of the Journal send us for its pages some original observation for publication? Will they use their personal influence with a professional neighbor or friend, to enlarge the list of subscribers, and will the profession regard the Journal as its own, and make it in every respect such as shall merit unqualified approval? It will continue to be conducted in its interest, to defend its rights, to endeavor to increase its influence and usefulness, opposing what is false, and presenting only what is believed to be the truth in medicine.

